# Clonal dissemination of linezolid-resistant *Staphylococcus capitis* with G2603T mutation in domain V of the 23S rRNA and the *cfr* gene at a tertiary care hospital in China

**DOI:** 10.1186/s12879-015-0841-z

**Published:** 2015-02-26

**Authors:** Wanqing Zhou, Dongmei Niu, Xiaoli Cao, Mingzhe Ning, Zhifeng Zhang, Han Shen, Kui Zhang

**Affiliations:** Department of Laboratory Medicine, Nanjing Drum Tower Hospital, the affiliated Hospital of Nanjing University Medical School, 321# Zhongshan Road, Gulou District, Nanjing, Jiangsu Province 210008 PR China; Department of Laboratory Medicine, Nanjing Jinling Hospital, the affiliated Hospital of Nanjing University Medical School, 305# East Zhongshan Road, Baixia District, Nanjing, Jiangsu Province 210002 PR China

**Keywords:** Linezolid, Resistance, *cfr* gene, 23S rRNA gene, *Staphylococcus capitis*

## Abstract

**Background:**

The present study aims to investigate the potential mechanism of linezolid-resistant *Staphylococcus capitis* (LRSC) isolates collected from our hospital.

**Methods:**

The susceptibilities of 5 *Staphylococcus capitis* isolates displaying resistance towards linezolid were determined by E-test. Polymerase chain reactions (PCRs) and DNA sequencing were used to investigate the potential molecular mechanism. Clonal relatedness between these strains was analyzed by pulsed-field gel electrophoresis (PFGE).

**Results:**

The MICs of linezolid on these 5 isolates were >256 μg/mL. The G2603T mutation was observed in the domain V of the 23S rRNA with *cfr* gene being also widely detected among these 5 strains. PFGE analysis displayed close genetic relatedness between these linezolid-resistant isolates.

**Conclusions:**

The emergence of LRSC isolates carrying G2603T mutation in the domain V of the 23S rRNA and harboring *cfr* gene in our hospital may pose a potential challenge to the public health.

## Background

Linezolid is the first member of an entirely new class of antibiotics that can inhibit bacterial protein synthesis by binding to the 50S subunit of the bacterial ribosome *via* interaction with the 23S rRNA of gram-positive bacteria [1]. Based on the unique mechanism of action, it is highly effective in the treatment of serious infections caused by antibiotic resistant gram-positive bacteria such as methicillin-resistant *Staphylococcus aureus* and vancomycin-resistant enterococci [1]. At the introduction of linezolid, it was claimed that there would be no cross-resistance to linezolid and resistance would be rare and difficult for the bacteria to develop. However, linezolid-resistant staphylococci and enterococci have been increasingly reported in recent years [2-5], since the first case report on the emergence of linezolid-resistant MRSA in North America in 2001 [6].

The mutations at the central loop of the domain V region on the 23S rRNA gene have been well recognized as the main mechanism mediating resistance to linezolid [6,7]. The acquisition of the chloramphenicol-florfenicol resistance (*cfr*) gene encoding the 23S rRNA methyltransferase and modifying adenosine at position 2503 in the 23S rRNA has been frequently reported [2]. In addition, mutations or deletions in genes encoding the 50S ribosomal subunit proteins L3 or L4 have also played important role [8-10]. Up to date, the linezolid-resistant coagulase-negative *Staphylococcus* (LRCoNS) isolates have frequently emergenced among patients in many countries including North America (USA, Mexico), South America (Brazil), Europe (Greece, Spain, Italy, France, and Ireland), and Asia (India) [2,5,7,8]. However, such strains have rarely been reported in China [3,4,11], especially for the linezolid-resistant *Staphylococcus capitis* (LRSC) isolates [12]. In the present study, we report the emergence and potential clonal dissemination of 5 LRSC isolates among a tertiary care hospital of China.

## Methods

### Bacterial strains and data collection

A total of 29 LRSC isolates were continuously recovered from the blood (n = 28) and catheter (n = 1) from 5 hospitalized patients in 2 different wards at Nanjing Drum Tower Hospital (Nanjing, China) between September, 2012 and February, 2014. Among them, 5 LRSC isolates recovered from the blood (n = 4) and catheter (n = 1) were further analyzed in this study. And 4 linezolid-susceptible *S. capitis* (LSSC) isolates (SA01, SA02, SA03, SA04) from 2 wards during the same period were used as control. Blood culture was implemented according to the principle of double-sided bottles, the blood samples were processed using the BacT/Alert automated system (bioMerieux, France) and subcultured on Colombia plate containing 5% sheep blood (bioMerieux, France). Catheter tips were inoculated on Colombia blood agar by roll pate method. Coagulase-negative *Staphylococcus* were identified by colony morphology, gram staining, catalase testing and coagulase assays. The confirmation of *Staphylococcus capitis* was performed by using Vitek 2 Compact GP card (bioMerieux, France) combined with additional sequencing for the 16S ribosomal ribonucleic acid (16S rRNA) gene as described below. Clinical data such as the clinical features, laboratory results, and treatment were retrieved from the medical records department. The study protocol was approved by the Ethics Committee of Nanjing Drum Tower Hospital and written informed consent was obtained from all patients included in the study.

### 16S rRNA sequencing for species identification

Chromosomal DNA was extracted from overnight cultures of these isolates grown on Colombia plate containing 5% sheep blood (bioMerieux, France) using the Qiagen DNA mini kit (Qiagen, Hilden, Germany), according to the manufacturer’s instructions. Molecular identification of the strains was performed by PCR with primes of 5′-AGA GTT TGA TCM TGG CTC AG-3′ and 5′-TAC GGY TAC CTT GTT ACG ACT T-3′, which generated a 1.4- kilobase-pair (kbp) amplicon. Amplification was carried out using Ex Taq™ DNA polymerase (TaKaRa) according to the manufacturer’s instruction. The 25 μl reaction mixture for the PCR assays contained the following: 10 mM Tris/HCL (pH 8.3), 50 mM KCl, 1.5 mM MgCl_2_, 200 μM deoxynucleotide triphosphate and 25 pmol of each primer. Amplification conditions were as follows: 95°C for 5 min, followed by 30 cycles of 95°C for 30 s, 55°C for 30 s and 72°C for 1 min as well as 72°C for 10 min. PCR products were sequenced by using an ABI 3730XL fluorescence sequencer (Applied Biosystems, Foster City, Calif.) after they were purified by using the purification kit (Qiagen, Hilden, Germany).

### Antimicrobial susceptibility testing

Antimicrobial susceptibility testing was performed by using the disc diffusion tests (Oxoid) on Mueller Hinton agar (bioMerieux, France) according to Clinical and Laboratory Standards Institute guidelines [13], with the following antibiotics being included: penicillin (30 μg), ampicillin (10 μg), gentamicin (10 μg), ciprofloxacin (5 μg), levofloxacin (5 μg), chloromycetin (30 μg), tetracycline (30 μg), cefoxitin (30 μg), trimethoprim-sulfamethoxazole (25 μg), erythromycin (15 μg), clindamycin (15 μg), ofloxacin (5 μg), amikacin (30 μg), rifampicin (5 μg) and linezolid (30 μg). Minimum inhibitory concentrations (MICs) of vancomycin, teicoplanin, tigecycline, quinupristin/dalfopristin, clindamycin and linezolid were determined using CLSI agar dilution methodology and the MICs of linezolid on the 5 LRSC isolates was also determined by E-test (AB Biodisk, Solna, Sweden). *S. aureus* ATCC 25923 and 29213 were used as the quality controls in parallel.

### Molecular detection of resistance genes

Amplification for *cfr* gene, the domain V of the 23S rRNA gene and the genes encoding ribosomal proteins L3, L4 and L22 was implemented as previously described by Mendes *et al.* [8]. The amplicons were purified by using Qiagen DNA purification kit (Qiagen, Hilden, Germany) and subjected to sequencing. The sequence similarity was determined by using the BLAST program from the National Center for Biotechnology Information (http://blast.ncbi.nlm.nih.gov/Blast.cgi).

### Pulsed-field gel electrophoresis (PFGE)

PFGE was carried out according to the protocol described by Yang *et al*. [11] with the CHEF Mapper XA system (Bio-Rad). Briefly, genomic DNA was digested with *Sma-I* and electrophoresis was conducted at 6 V/cm for 22 h at 14°C, with initial and final pulses conducted for 3.0 and 40.0 s, respectively. The PFGE types were defined on the basis of DNA banding patterns according to the criteria of Tenover *et al*. [14].

## Results

### Clinical data

29 LRSC isolates were collected from these 5 patients. The number of samples taken from each patient and the no. of isolates taken has been showed in Table 1. However, we just took 5 strains including SA10106, SA13096, SA23062, SA20062, and SA11026 from these 5 patients respectively for further analysis in our study. As it was shown in Table 2, medical records showed that SA10106 and SA13096 out of the 5 LRSC isolates were isolated from different wards during the same time period, SA23062 and SA20062 from the same wards during the near time period. However, SA11026 was isolated in 2014, which was quite later than the previous 4 isolates. It’s noteworthy that 4 LRSC isolates were collected from ICU, only 1 from the department of infectious diseases. In addition, among the 5 patients carrying LRSC isolates, 3 ones received linezolid for treatment which lasted more than 13 days with the total dosage being above 7.8 g.

**Table 1:** **The number of samples taken from each patient and the corresponding strain codes**

**Patient**	**Ward**	**Strains isolated**	**Resource**	**Collection date (yy/mm/dd)**
1	ICU	SA10106	catheter of femoral vein	2012/9/10
		SA11055	blood	2012/9/11
		SA11056	blood	2012/9/11
		SA11057	blood	2012/9/11
		SA11058	blood	2012/9/11
2	IDD	SA13096	blood	2012/9/13
		SA13097	blood	2012/9/13
		SA13098	blood	2012/9/13
		SA13099	blood	2012/9/13
3	ICU	SA23062	blood	2013/6/23
		SA23063	blood	2013/6/23
		SA23064	blood	2013/6/23
		SA23065	blood	2013/6/23
		SA25077	blood	2013/6/25
		SA25078	blood	2013/6/25
		SA25079	blood	2013/6/25
		SA25080	blood	2013/6/25
4	ICU	SA20062	blood	2013/7/20
		SA20063	blood	2013/7/20
		SA20064	blood	2013/7/20
		SA20065	blood	2013/7/20
		SA22101	blood	2013/7/22
		SA22102	blood	2013/7/22
		SA22103	blood	2013/7/22
		SA22104	blood	2013/7/22
5	ICU	SA11026	blood	2014/2/11
		SA11027	blood	2014/2/11
		SA11028	blood	2014/2/11
		SA11029	blood	2014/2/11

Note: ICU: intensive care unit; IDD: the department of infectious diseases.

**Table 2:** **Clinical and resistant characteristics of clinical linezolid-resistant** ***Staphylococcus capitis***

**Strain designation**	**Ward**	**Resource**	**Isolation(yy/mm/dd)**	**Linezolid usage (total dose/days)**	**MIC ( μg/mL)**				***cfr***	**Mutations in domain V of the 23S rRNA gene**
					**LZD**	**VAN**	**Q/D**	**CLI**		
SA10106	ICU	catheter	2012/9/10	7.8 g/13 days	>256	1	>=8	2	+	G2603T
SA13096	IDD	blood	2012/9/13	–	>256	<=0.5	>=8	2	+	G2603T
SA23062	ICU	blood	2013/6/23	8.4 g/14 days	>256	1	>=8	2	+	G2603T
SA20062	ICU	blood	2013/7/20	13.2 g/22 days	>256	<=0.5	>=8	2	+	G2603T
SA11026	ICU	blood	2014/2/11	–	>256	<=0.5	>=8	2	+	G2603T

Note: ICU: intensive care unit; IDD: the department of infectious diseases; LZD: linezolid; VAN: vancomycin; Q/D: quinupristin/dalfopristin; CLI: clindamycin; −, no linezolid treatment.

### Molecular identification of strains

Further 16S rRNA sequencing and analysis confirmed that all the 5 LRSC isolates and the 4 LSSC isolates belonged to *S. capitis*, which was in accordance with conventional method. The phylogenetic tree constructed by neighbor-joining method showed the position of our isolates with respect to *S. capitis* strain BQEP2-01d with the accession number of FJ380956.1.

### Antimicrobial susceptibility testing

Similar multidrug-resistant phenotypes were observed in the 5 LRSC isolates (Table 2). All the 5 isolates were resistant to linezolid indicated both by disk diffusion testing and by the E-test. However, the MICs of vancomycin and teicoplanin were ≤ 2 μg/mL, and the MICs of tigecycline were 0.25 μg/mL. In contrast, all the LSSC isolates showed susceptible to the antimicrobial agents tested which are quite different from the susceptibilities of LRSC isolates.

### Genetic analysis and resistance-related genes of LRSC isolates

**Figure 1:**
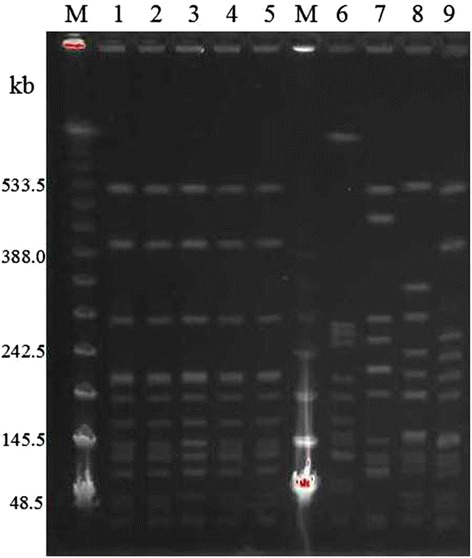
**PFGE profiles of** ***Staphylococcus capitis*** **obtained with** ***Sma-I*** **.** M, λ molecular marker; Line 1, SA10106; Line 2, SA13096; Line 3, SA23062; Line 4, SA20062; Line 5, SA11026; Line 6, SA01; Line 7,SA02; Line 8, SA03; Line 9, SA04.

PCR mapping and sequencing results showed that G2576T, the most common mutation in domain V of the 23S rRNA gene [10], was not be detected in all of the 5 LRSC isolates*.* However, a new mutation, G2603T, which had not been previously reported in *S. capitis*, was observed in these 5 isolates with the *cfr* gene being simultaneously prevalent among them (Table 2), while none of the 4 LSSC isolates carried *cfr* gene and had mutations in the 23S rRNA. Moreover, the mutations in the L3, L4 or L22 were not detected among LRSC isolates in our current study. The genetic relationship between these LRSC strains (Figure 1) revealed a clonal dissemination of LRSC isolates as displayed by the identical *Sma-I* digestion patterns.

## Discussion

With the frequent use of linezolid in clinical treatment, the LRCoNS strains have been increasingly isolated from health care setting. In this study, we report the linezolid resistance mediated by the G2603T mutations accompanied the prevalence of *cfr* genes in LRSC isolates and a clonal dissemination of LRSC strain among 5 patients at a tertiary-care hospital between September 2012 and February 2014 in Nanjing, Jiangsu province of China.

As it has been previously reported, LRCoNS isolates only kept sensitivities to vancomycin, teicoplanin, tetracycline, tigecycline and rifampicin *in vitro* [2]. Previous report showed that LRCoNS strains were isolated with the mean time of 11.0 ± 8.0 days after patients were treated with linezolid, although in a few cases, the resistant strains were acquired as a result of cross-infection [2]. This may provide further evidence to the proposal that the linezolid usage is an independent risk factor for development of LRCoNS strains [5]. This has been partly evidenced by the founding in our study that 3 out of 5 patients carrying LRSC isolates received linezolid therapy at least for 13 days with a total dose of 7.8 g before the isolation of LRSC. Taking into account of the isolation of the same LRSC clone among these 5 patients and the time periods of the strains being isolated, we speculate that it may be a clonal dissemination of LRSC strain [15].

Generally, *cfr* methylation confers resistance to 5 classes of 50S ribosomal subunit-targeted antibiotics defined by the PhLOPS_A_ phenotype, including phenicols, lincosamides, oxazolidinones, pleuromutilins, and streptogramin A [10]. And *cfr*-carrying staphylococci therefore display multi-drug resistant phenotype, which is in agreement with the resistance profiles of our isolates (Table 2). Noteworthily, the *cfr* gene has been found primarily on plasmids, which can be transferred between staphylococci. In a recent paper, *cfr* gene has been described to play a decisive role in mediating resistance to linezolid [11]. The identification of a high incidence of *cfr* among our LRSC strains indicates a probability of horizontal gene transfer, which alerts us the necessity of strengthening the implementation of infection and control measures.

Additionally, previous studies demonstrated that _△_His 146 and Gly155Arg in L3 in laboratory-derived *S. aureus* and mutations within a conserved region of the L4 protein (_63_KPWRQKGTGRAR_74_) have been associated with cross-resistance to linezolid in *Streptococcus pneumoniae*, *S. aureus* and *Clostridium perfringes* [9,16,17]. Nevertheless, such resistance mechanisms were not detected in ribosomal proteins L3, L4 or L22 of the 5 LRSC isolates in our study. Instead, we observed a novel mutation in the 23S rRNA gene [G(2603)T] which is quite different from the frequent mutations in the central loop of domain V of the 23S rRNA in most linezolid-resistant isolates, such as enterococci and staphylococci. As far as we know, G2528U, G2576U, and G2505A have been identified in linezolid-resistant enterococci, G2447U and G2576U in linezolid-resistant *S. aureus* [2,10], and C2534T, G2447T, G2576T, T2504A, C2109T and G2474T in the increasing LRCoNS which was correlated with nosocomial transmission [2]. It’s worthy to note that the most common mutation in domain V of the 23S rRNA gene, G2576T, was also not detected in our 5 LRSC isolates, whereas, G2603T was detected in all these 5 isolates. And the presence of G2603T mutation has been reported to be crucial for conferring resistance to linezolid in *S. epidermidis* [18] and *S. hominis subsp. hominis* [19]. However, there has been no report on the G2603T mutation in *S. capitis* isolate so far. In addition, it was found that the degree of linezolid resistance is associated with the number of mutations occurring in the copies of the 23S rRNA coding gene [20]. In our study, the entire 23S rRNA gene of the LRSC isolates were not sequenced for all copies, it is possible that other resistance mechanisms may co-exist in these strains.

*S. capitis* has been involved in biofilm related infections such as endocarditis, urinary tract infection, and catheter-related bacteremia. Hospital outbreaks caused by LRCoNS have been reported in the United States, Ireland, and Spain [21]. It has also been reported that *S. capitis* is emerging as an opportunistic pathogen in newborn babies [22]. The presence of LRSC isolates in healthcare setting therefore has been a major concern based on the bacteremia and catheter-related infections that they frequently caused. Since resistance has most commonly occurred in most patients undergoing long-term linezolid therapy [2], and the heavy use of linezolid in the patients may create substantial selection pressure in favor of linezolid-resistant isolate [5]. Thus, prudent use of linezolid in clinical treatment becomes very important.

## Conclusions

In conclusion, the prevalence of *cfr* gene and the existence of G2603T mutation together lead to the resistance of our strains to linezolid. In addition, the clonal dissemination of such LRSCs may pose a potential threat to the public health.

## Abbreviations

LRSC: Linezolid-resistant *S. capitis*
MICs: Minimum inhibition concentrations
PFGE: Pulsed-field gel electrophoresis
LRCoNS: Linezolid-resistant coagulase-negative *Staphylococcus*
LSSC: Linezolid-susceptible *S. capitis*
